
JOURNAL INFORMATION
==============================
NLM Title Abbreviation: ScientificWorldJournal
Journal ID: ScientificWorldJournal
NLM Journal ID: 101131163
Journal ID: TSWJ

The Scientific World Journal

ISSN: 2356-6140
EISSN: 1537-744X
Publisher: Wiley

ARTICLE INFORMATION
==============================
PMCID: PMC5823222
PMID: 19578705
DOI: 10.1100/tsw.2009.71
Article ID: 570123
Article version: 1
Subjects: Editorial

Update on Lower Urinary Tract Symptoms

Badlani Gopal H. *
Department of Urology, Wake Forest University Baptist Medical Center, Winston-Salem, NC, USA
*Gopal H. Badlani: gbadlani@wfubmc.edu
Publication date: 2009
Electronic publication date: 2009 Jun 30
Volume: 9
First page: 499
Last page: 500
Received 2009 Jun 10; Revised 2009 Jun 23; Accepted 2009 Jun 23
Copyright: Copyright © 2009 Gopal H. Badlani.
Copyright year: 2009
License: This is an open access article distributed under the Creative Commons Attribution License, which permits unrestricted use, distribution, and reproduction in any medium, provided the original work is properly cited.
License URL: https://creativecommons.org/licenses/by/3.0/

==============================
Editorial
Special Issue: Update on Lower Urinary Tract Symptoms
*TheScientificWorldJOURNAL* (2009) 9, 499–500
TSW Urology
ISSN 1537-744X; DOI 10.1100/tsw.2009.71

TheScientificWorldJOURNAL
www.thescientificworld.com

### Update on Lower Urinary Tract Symptoms

Gopal H. Badlani

*Department of Urology, Wake Forest University Baptist Medical Center, Winston-Salem, NC*

**E-mail**: gbadlani@wfubmc.edu

*Received June 10, 2009; Revised June 23, 2009; Accepted June 23, 2009; Published June 30, 2009*

---

Management of incontinence is forever changing, evolving as our understanding of the pathophysiology improves. It sometimes feels like a sand castle, as the principles of therapy of yesterday are replaced by new modalities, without leaving a trace of treatments in which we so firmly believed.

This special issue of *TheScientificWorldJOURNAL: TSW Urology* was aimed at new treatments, starting with terminology[1]. Karl Eric Anderson has been involved with the ICS and the terminology committee for a number of years and, thus, along with Dr. Vishwajit, has the historical perspective to question the new terminology. Accurate descriptions are immensely helpful in order to define and treat incontinence and associated voiding dysfunction.

The assessment of the symptoms and the need for urodynamics is a controversial topic, and it is dissected by Fletcher and Lemack[2]. The cost implication of testing vs. a poor outcome due to incorrect diagnosis is debated. Although there are no set guidelines, the article will help in the understanding of the problem.

Therapy in the elderly, especially the cognitive impairment with anticholinergic load, is a point of controversy. Patel and colleagues[3] carefully looked at the peer-reviewed data to see if the "marketing hype" had any scientific validity.

Minimally invasive therapy is the buzzword for treating stress incontinence and prolapse. The use of injectable materials and devices to bring about coaptation was a concept popularized by McGuire and colleagues[4]. A number of agents were developed to improve upon the initial use of Teflon, including the periurethral balloons. Crivellaro and Smith[5] methodically looked at the experience, including their own, to update us on the current use of these techniques and their place in the armamentarium to treat incontinence.

Biomaterials are increasingly used to correct pelvic organ prolapse and stress urinary incontinence. The scientific basis for use of these materials lies in the biochemical defect that exists in patients with pelvic organ prolapse, as well as the clinical recurrence rate when biomaterials are not used. The evidence is still largely level 3, but randomized trials data are around the corner. Dr. Amrute[6] has been my associate in developing these concepts.

The use of biomaterials, when combined with kits, decreases the morbidity to the patient and standardizes the surgical procedures. Drs. Moore and Miklos[7] have wide experience in the use of these kits and share the nuances of techniques, lessons learned, as well early outcomes of multicenter trials. The cost implications of using these kits are a public health concern, counterbalanced by individual patient decrease in morbidity and recurrence rate.

The material, technique (surgeon), and host (patient) form a three-legged stool on which rests the outcome. So when complications of the procedure occur, is it the product, the technique, or the host environment that cause the question detailed by Dr. Kobashi[8]. She has had the clinical experience with a variety of materials as well as the database to track the long-term outcomes.

©2009 with author.
Published by TheScientificWorld; www.thescientificworld.com

The world of urology has been taken over by the alien "da Vinci robot". Dr. Hemal[9] is a true wizard with the use of this tool and, along with Dr. Singh, shows us how it could be used to treat complex pelvic cases, including fistulas, and its possible future role in reconstructive surgery.

Bladder overactivity has seen a sea of change, from terminology (e.g., OAB, overactive bladder) to a plethora of drugs on the market. There is, however, a proportion of patients who are refractory to these treatments. The advances in neuromodulation described in this issue by Hussain and Harrison[10] and intravesical therapy with botulinum toxin detailed by Morrisroe and Chancellor[11] have spared many patients the need for bladder augmentation or other invasive procedures.

It is never easy to project what will be new and exciting, yet have clinical relevance, as well as some evidence of success to put together a special issue on incontinence that offers meaningful information. I am thankful to my colleagues for making this issue possible in a timely manner, for their insightful commentary, and to the unnamed reviewers who took the time to review these manuscripts.

### REFERENCES

1. Vishwajit, S. and Andersson, K.-E. (2009) Terminology of lower urinary tract symptoms. Helpful or confusing? *TheScientificWorldJOURNAL:* TSW Urology **9**, 17–22. DOI 10.1100/tsw.2009.9.
2. Fletcher, S.G. and Lemack, G.E. (2008) Clarifying the role of urodynamics in the preoperative evaluation of stress urinary incontinence. *TheScientificWorldJOURNAL:* TSW Urology **8**, 1259–1268. DOI 10.1100/tsw.2008.159.
3. Patel, B., Bavendam, T., and Badlani, G. (2009) Use of antimuscarinics in the elderly. *TheScientificWorldJOURNAL:* TSW Urology **9**, 459–465. DOI 10.1100/tsw.2009.55.
4. McGuire, E.J. (2006) Urethral bulking agents. *Nat. Clin. Pract. Urol.* **3,** 234–235. DOI 10.1038/ncpuro0478.
5. Crivellaro, S. and Smith, J.J., III (2009) Minimally invasive therapies for female stress urinary incontinence: the current status of bioinjectables/new devices (adjustable continence therapy, urethral submucosal collagen denaturation by radiofrequency). *TheScientificWorldJOURNAL:* TSW Urology **9**, 466–478. DOI 10.1100/tsw.2009.53.
6. Amrute, K.V. and Badlani, G.H. (2009) The science behind biomaterials in female stress urinary incontinence surgery. *TheScientificWorldJOURNAL:* TSW Urology **9**, 23–31. DOI 10.1100/tsw.2009.7.
7. Moore, R.D. and Miklos, J.R. (2009) Vaginal mesh kits for pelvic organ prolapse, friend or foe: a comprehensive review. *TheScientificWorldJOURNAL:* TSW Urology **9**, 163–189. DOI 10.1100/tsw.2009.19.
8. Kobashi, K.C. (2009) Management of erosion of graft materials in pelvic floor reconstruction. *TheScientificWorldJOURNAL:* TSW Urology **9**, 32–36. DOI 10.1100/tsw.2009.2.
9. Singh, I. and Hemal, A.K. (2009) Role of robot-assisted pelvic surgery. *TheScientificWorldJOURNAL:* TSW Urology **9**, 479–489. DOI 10.1100/tsw.2009.54.
10. Hussain, Z. and Harrison, S.C.W. (2007) Neuromodulation for lower urinary tract dysfunction — an update. *TheScientificWorldJOURNAL:* TSW Urology **7,** 1036–1045. DOI 10.1100/tsw.2007.173
11. Morrisroe, S.N. and Chancellor, M.B. (2007) Botulinum toxin use in the lower urinary tract. *TheScientificWorldJOURNAL:* TSW Urology **7,** 808–817. DOI 10.1100/tsw.2007.150.

**This article should be cited as follows:**

Badlani, G. (2009) Update on lower urinary tract symptoms. *TheScientificWorldJOURNAL:* TSW Urology **9**, 499–500. DOI 10.1100/tsw.2009.71.
